# Distinct immunophenotypic and clinical features of TP53-mutated acute myeloid leukemia: high CD34/CD41 expression and lower leukocyte counts

**DOI:** 10.1007/s00277-026-07001-4

**Published:** 2026-04-21

**Authors:** Songyi Park, Sang-A Kim, Sheehyun Kim, Seon Young Kim, Ji Yun Lee, Ja Min Byun, Sang Mee Hwang, Jeong-Ok Lee, Youngil Koh, Junshik Hong, Yoon Hwan Chang, Hyun Kyung Kim, Soo-Mee Bang, Inho Kim, Sung-Soo Yoon, Dong-Yeop Shin

**Affiliations:** 1 Division of Hematology and Medical Oncology, Department of Internal Medicine, Seoul Metropolitan Government Seoul National University (SMG-SNU) Boramae Medical Center, Seoul, 07061 Republic of Korea; 2 Division of Hematology and Medical Oncology, Department of Internal Medicine, Seoul National University Bundang Hospital, Seongnam-si, 13620 Gyeonggi-do Republic of Korea; 3 Center for Precision Medicine, Seoul National University Hospital, Seoul, 03080 Republic of Korea; 4 Center for Medical Innovation, Clinical Research Institute, Seoul National University Hospital, Seoul, 03080 Republic of Korea; 5 Department of Laboratory Medicine, Seoul National University Hospital, Seoul, 03080 Republic of Korea; 6 Division of Hematology and Medical Oncology, Department of Internal Medicine, Seoul National University Hospital, Seoul, 03080 Republic of Korea; 7 Cancer Research Institute, Seoul National University College of Medicine, Seoul, 03080 Republic of Korea; 8 Department of Laboratory Medicine, Seoul National University Bundang Hospital, Seongnam-si, 13620 Gyeonggi-do Republic of Korea

**Keywords:** Acute myeloid leukemia, *TP53* mutation

## Abstract

This study investigates the clinical and molecular characteristics of *TP53*-mutated acute myeloid leukemia (AML), associated with a poor prognosis. A retrospective analysis was conducted on 336 AML patients, focusing on the distinctions between *TP53*-mutated and non-mutated cases. Our findings reveal that *TP53* mutations are linked to a higher proportion of leukemic blasts positive for the primitive stem cell markers CD34 and CD41 (with dual positive: 12.5% vs. 1.3%, *p* < 0.001), suggesting a more stem/progenitor cell-like nature. Patients with *TP53*-mutated AML exhibited lower white blood cell counts in peripheral blood (*p* = 0.016) and reduced blast proportion in bone marrow (with a median of 39.6% in *TP53*-mutated AML and 56.9% in *TP53* wild-type AML, *p* = 0.01) at diagnosis. *TP53*-mutated AML was frequently found in therapy-related AML cases and occurred with fewer concurrent genetic mutations compared to non-mutated AML. In terms of outcomes, *TP53* mutations corresponded with significantly lower composite complete response rates and shorter overall and progression-free survival. This study highlights the distinct clinical, molecular, and immunophenotypic features of TP53-mutated AML and emphasizes the need for improved therapeutic strategies for this high-risk subtype.

**Supplementary Information:**

The online version contains supplementary material available at 10.1007/s00277-026-07001-4.

## Introduction

Acute myeloid leukemia (AML) is an aggressive hematologic malignancy with an incidence of 4 per 100,000 annually and a median diagnosis age of 70 years, resulting in 85,000 deaths globally each year [1, 2]. The incidence of AML increases with age and remains higher in developed countries [3]. The pathophysiology of AML is not yet fully understood. However, recent advancements in next-generation sequencing (NGS) have significantly enhanced our comprehension of the genomic architecture of AML. Numerous studies have identified several recurrent mutations that play critical roles in the disease’s biology, response to therapy, and overall prognosis [4, 5]. Specifically, mutations in genes such as *CEBPA*, *NPM1*, *FLT3*, *RUNX1*, *ASXL1*, and *TP53* have been found to be strongly associated with prognosis. These genetic alterations have been integrated into the recent European Leukemia Net (ELN) risk stratification guidelines and World Health Organization (WHO) classifications, underscoring their importance in predicting disease outcomes and guiding therapeutic decisions [6, 7].

*TP53* is a critical tumor suppressor gene mutated in approximately 50% of human cancers, which, when functioning properly, activates stress-induced apoptosis and cell cycle arrest through the regulation of numerous genes, including pro-apoptotic proteins. This mutation undermines p53’s ability to control these processes, contributing to cancer progression and resistance to anticancer treatment [8]. Most de novo cases of AML exhibit unaltered *TP53* alleles, while *TP53* mutations are frequently found in therapy-related AML (t-AML) and AML with myelodysplasia-related changes (AML-MRC). These mutations are linked to complex cytogenetic abnormalities, advanced age, chemoresistance, and poor outcomes, highlighting the need for improved treatment strategies, especially given recent advances in targeted therapies [9–12].

Previous studies have suggested that *TP53*-mutated AML is frequently associated with lower leukocyte counts and higher CD34 expression. However, the broader immunophenotypic characteristics of TP53-mutated AML and their clinical implications remain incompletely defined [13, 14]. This study aims to provide a detailed characterization of *TP53*-mutated AML, focusing on its distinct clinical and molecular features, while also exploring the potential existence of characteristics in patients who exhibit a good prognosis despite carrying a *TP53* mutation. We will analyze the characteristics and clinical prognosis of patients with *TP53*-mutated AML according to the specific details of their mutations.

## Methods

### Study design and population

The study was designed as a retrospective analysis involving a total of 336 patients diagnosed with AML between 2017 and 2023 at Seoul National University Hospital (SNUH) and Seoul National University Bundang Hospital (SNUBH), with 192 patients from SNUH and 144 patients from SNUBH. Eligible patients were identified through the electronic database of these hospitals. To qualify for inclusion in the study, patients had to undergo immunophenotyping and NGS panel testing at the time of their AML diagnosis. Patients were classified according to the ELN 2017 risk classification based on cytogenetic and molecular abnormalities.

### Immunophenotype

Bone marrow (BM) samples or peripheral blood samples at initial diagnosis were used for immunophenotyping and NGS panels. Immunophenotyping was performed using flow cytometry with fluorochrome-conjugated monoclonal antibodies against hematopoietic lineage markers including CD2, CD3, CD4, CD5, CD6, CD7, CD8, CD10, CD13, CD14, CD19, CD20, CD33, CD34, CD41, CD56, CD64, and CD117. Data acquisition was performed using a BD FACSCanto II flow cytometer (BD Biosciences, San Jose, CA, USA). Surface antigen expression was evaluated in blast populations identified based on CD45 expression and side scatter characteristics. We analyzed the characteristics by dividing them into two groups according to the expression level of the markers. Following the EGIL criteria, we interpreted the positivity of blast cells for a particular marker using a cutoff of 20% for surface markers and 10% for cytoplasmic markers, classifying expressions that met or exceeded these percentages as positive for grouping [15].

### Targeted sequencing and cytogenetic analyses

Targeted sequencing was performed using anchored multiplex polymerase chain reaction (PCR) enrichment based on NGS. At SNUH, NGS was conducted on 87 genes from 2017 to 2020, and from 2021 onward, sequencing was performed on 103 specific genes. At SNUBH, NGS was performed on 38 specific genes. To exclude bias due to the different gene sets, only genes included in both gene panels from two hospitals were used for the final analysis in this study. (Supplement Table S1) [16]. NGS was performed on bone marrow aspirates or peripheral blood samples using the Oncomine Myeloid Research Assay on the Ion S5 XL platform (Thermo Fisher Scientific, Waltham, MA, USA) or a customized myeloid gene panel on the NextSeq550Dx platform (Illumina, San Diego, CA, USA). Sequencing data were analyzed using the Clinical Genomics Workspace (PierianDX Inc., St. Louis, MO, USA) with institutional bioinformatics pipelines. The average depth of coverage exceeded 200×, and variants with a variant allele frequency (VAF) ≥ 2% were considered for analysis. Common single nucleotide variants and insertion/deletion mutations observed at ≥ 0.1% frequency in population databases such as gnomAD were filtered out to exclude potential germline variants.

### Statistics

Statistical analyses of clinical parameters were performed using IBM SPSS Statistics version 25 (SPSS, Chicago, IL, USA) and RStudio. The chi-square test and logistic regression analysis were used to investigate the association of clinical variables. To account for multiple comparisons in the analyses of immunophenotypic markers and co-occurring mutations, false discovery rate correction using the Benjamini–Hochberg (BH) method was applied. The Kaplan-Meier method was used to obtain estimated survival results and comparisons were made using log-rank tests. Overall survival (OS) was calculated as the time from diagnosis to death from any cause or the last follow-up in survivors. Progression-free survival (PFS) was defined as the time from diagnosis until relapse or death from any cause. Two-sided *p* values < 0.05 were considered statistically significant.

## Results

### Patients’ characteristics

**Fig. 1:**
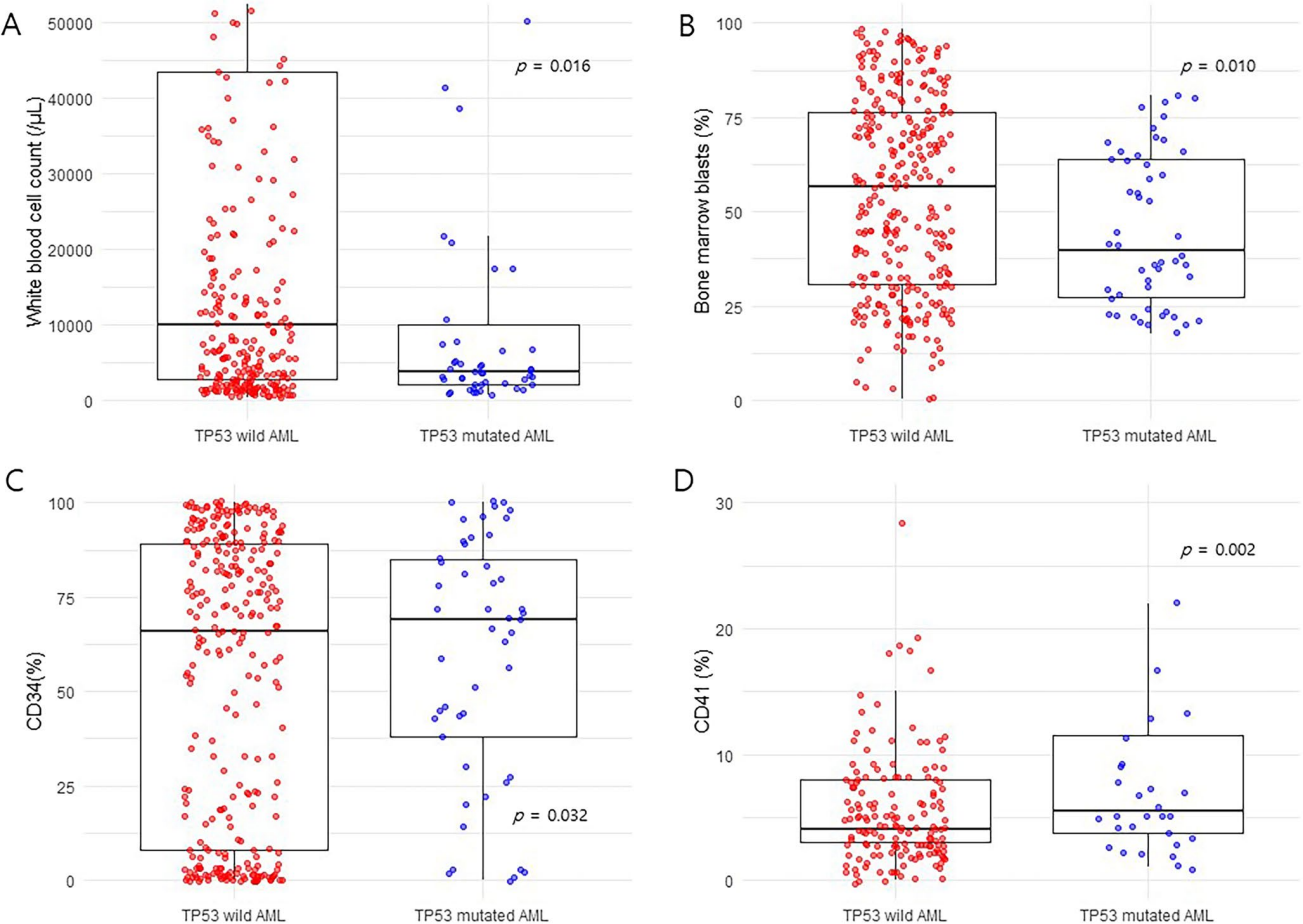
*Difference of baseline characteristics according to *TP53* mutation. (**A**) White blood cell counts (/µL). (**B**) Bone marrow blasts (%). (**C**) CD34. (**D**) CD41. CD: cluster of differentiation

Patients’ characteristics are described in Table 1. The characteristics were organized based on the presence or absence of *TP53* mutations. Among the cohort, 55.4% of the patients were male, while 44.6% were female; however, these gender distributions did not show any significant correlation with *TP53* mutation status. In the laboratory results at the time of diagnosis, it was observed that patients with *TP53*-mutated AML had significantly lower white blood cell (WBC) count and peripheral blasts compared to those with non-mutated AML (median WBC counts were 10,100/µL in patients with *TP53* wild-type AML and 3,835/µL in patients with *TP53*-mutated AML, respectively, *p* = 0.016, as shown in Fig. 1A). In addition, the percentage of bone marrow blasts was significantly lower in *TP53*-mutated AML, with a median of 39.6% (33.0-55.1), compared to 56.9% (48.7–62.0) in the non-mutated group (*p* = 0.010, as shown in Fig. 1B). Similarly, the percentage of peripheral blood blasts showed a trend toward reduction, with a median of 19.5% (7.0–24.0) in *TP53*-mutated AML versus 21.0% (16.0–26.0) in non-mutated AML, although the difference did not reach statistical significance (*p* = 0.055) (see Supplementary Fig. 1).

**Table 1:** *Patient characteristics

	N (%)	Total (*N* = 336)	*TP53* wild-type (*N* = 286)	*TP53* mutation (*N* = 50)	*p* value
Age, y (median, range)		62 (18–88)	61.5 (18–88)	62 (23–84)	0.290
Sex	Male	186 (55.4)	155 (54.2)	31 (62.0)	0.306
	Female	150 (44.6)	131 (45.8)	19 (38.0)	
Laboratory results at diagnosis (median, range)	White blood cell (/µL)	7,870 (400 − 302,010)	10,100 (400 − 302,010)	3,835 (730 − 130,980)	0.016
	Hemoglobin (g/dL)	8.5 (3.3–13.3)	8.5 (3.3–13.3)	8.05 (6.3–13.0)	0.570
	Platelet (/µL)	62,000 (4,000–1,356,000)	63,000 (4,000–1,356,000)	52,000 (6,000-659,000)	0.983
	Lactate dehydrogenase (U/L)	372 (36-14254)	372 (53-2941)	356 (36-14254)	0.019
	Bone marrow cellularity (%)	85.0 (80.0–85.0)	85.0 (80.0–85.0)	85.0 (70.0–90.0)	0.753
	Peripheral blast (%)	21.0 (17.0-24.5)	21.0 (16.0–26.0)	19.5 (7.0–24.0)	0.055
	Bone marrow blast (%)	52.9 (46.6–59.4)	56.9 (48.7–62.0)	39.6 (33.0-55.1)	0.010
	ΔBlast (BM-PB) (%)	19 (-2.3-84.0)	18.9 (15.8–22.0)	20.4 (14.5–26.3)	0.988
Etiology	De novo	257 (76.5)	226 (79.0)	31 (62.0)	0.020
	Secondary	79 (23.5)	60 (21.0)	19 (38.0)	
ENL 2017 risk	Favorable	78 (24.7)	78 (29.3)	0	< 0.001
	Intermediate	95 (30.1)	95 (35.7)	0	
	Adverse	143 (45.3)	93 (35.0)	50 (100)	
Karyotype	Normal karyotype	137 (41.4)	130 (46.1)	7 (14.3)	< 0.001
	Complex karyotype	51 (15.4)	16 (5.7)	35 (71.4)	< 0.001
	Monosomal karyotype	9 (2.7)	6 (2.1)	3 (6.1)	0.113
	Cytogenetic abnormalities not classified as favorable or adverse	84 (25.4)	81 (28.7)	3 (6.1)	< 0.001

*N* number; *ELN* European leukemia net

The lactate dehydrogenase (LDH) levels also tended to be lower in *TP53*-mutated AML patients (*p* = 0.019). Regarding etiology, acute myeloid leukemia (AML) with TP53 mutations was more frequently associated with secondary AML compared to de novo AML, with rates of 62.0% and 38.0%, respectively. In contrast, *TP53* wild-type AML showed a markedly different distribution, with 79.0% classified as de novo and only 21.0% as secondary AML (*p* = 0.020). According to the European Leukemia Net (ELN) 2017 risk stratification, *TP53* is included as a risk factor and classified as adverse. In addition, a complex karyotype was statistically more prevalent in *TP53*-mutated AML patients, with a rate of 71.4%, compared to only 5.7% in *TP53* wild-type AML patients (*p* < 0.001).

### Immunophenotype according to *TP53* mutation

Immunophenotype analyses categorized aberrantly expressed surface markers as B cell-, T cell-, NK cell-, or T/NK cell-markers; there appeared to be no significant correlation between *TP53* mutation status and the pattern of aberrant expression according to the cell lineages (Table 2). Looking into the immunophenotypic profiles by each single marker, cytCD22 and CD3 appeared to be positive in a small number of patients with *TP53*-mutated AML; however, the proportion of positive patients for each marker was small, and other markers of B and T cell lineages were not different between *TP53*-mutated and non-mutated patients. The positivity of CD34, a well-known hematopoietic stem/progenitor marker, was higher in patients with *TP53*-mutations than in patients without *TP53* mutations (83.7% vs. 68.6%, *p* = 0.032, as shown in Fig. 1C), although this association did not remain statistically significant after BH correction for multiple comparisons. This finding suggests that *TP53*-mutated AML cells may harbor more primitive nature within the hematopoietic hierarchy, considering a previous seminal paper demonstrating CD34 as one of the 17- leukemic stem cell gene stemness scores [17]. Another interesting finding is that CD41, known as a megakaryocyte and platelet marker was also relatively enriched in *TP53*-mutated AML compared to *TP53* wild-type AML (15.6% vs. 2.5%, *p* = 0.002, as shown in Fig. 1D), and this association remained statistically significant after BH correction for multiple testing (false discovery rate (FDR)-adjusted *p* = 0.01). Several studies suggest that CD41, the glycoprotein IIb/IIIa antigen is widely expressed in the hematopoietic system outside the megakaryocytic lineage. Human CD34 + CD41+ cell subsets contains both myeloid and lymphoid potentialities with long-term culture initiating cells [18, 19], CD41 has also studied to be a potential marker of definitive hematopoietic progenitor in the mouse embryo [20]. Another hematopoietic stem/progenitor marker, CD117 was not expressed differently between *TP53*-mutated and -wild AML groups (CD117 positive patients 89.8% in *TP53*-mutated vs. 86.6% in *TP53* wild-type, *p* = 0.535) (Supplementary Table S2). Taken together, *TP53*-mutated AML cells showed enrichment of primitive stem/progenitor markers, particularly CD41 expression, compared with TP53 wild-type AML cells.

**Table 2:** *Profiles of immunophenotype and gene mutations according to the *TP53* mutation

		Total (*N* = 336)	*TP53* wild-type (*N* = 286)	*TP53* mutation (*N* = 50)	*p* value	FDR-adjusted *p*
Aberrantly expressed cell lineage by Immunophenotype	B cell lineage	27 (8.0)	25 (8.7)	2 (4.0)	0.255	0.36
	T cell lineage	13 (3.9)	9 (3.1)	4 (8.0)	0.101	0.18
	NK cell lineage	64 (19.0)	56 (19.6)	8 (16.0)	0.552	0.64
	T/NK cell lineage	66 (19.6)	57 (9.9)	9 (18.0)	0.751	0.75
	CD34	235 (70.8)	194 (68.6)	41 (83.7)	0.032	0.07
	CD41	9 (4.8)	4 (2.5)	5 (15.6)	0.002	0.01
	Both CD34 & CD41	6 (3.2)	2 (1.3)	4 (12.5)	< 0.001	0.01
Gene mutations	*ASXL1*	54 (16.1)	49 (17.1)	5 (10.0)	0.205	0.2
	*BCOR*	14 (4.2)	14 (4.9)	0	0.110	0.15
	*CEBPA*	33 (9.8)	33 (11.5)	0	0.011	0.04
	*FLT3*	18 (5.4)	18 (6.3)	0	0.068	0.12
	*IDH1*	22 (6.5)	22 (7.7)	0	0.042	0.11
	*KRAS*	18 (5.4)	18 (6.3)	0	0.068	0.2
	*NPM1*	64 (19.0)	62 (21.7)	2 (4.0)	0.003	0.02
	*RUNX1*	46 (13.7)	40 (14.0)	6 (12.0)	0.706	0.71

*FDR* false discovery rate, *N* number

### Concurrent mutations in *TP53*-mutated AML

Examination of the NGS panel revealed that *TP53* mutations often occurred without concurrent mutations in other genes. Specifically, NGS data focusing on seven recurrently mutated genes in AML indicated that patients with *TP53*-mutated AML had fewer concurrent mutations compared to those without *TP53* mutations. *NPM1*,* IDH1*, and *CEBPA* mutations were detected only in *TP53* wild-type patients in most cases (frequencies of *NPM1*, *IDH1*, and *CEBPA* in *TP53* wild-type vs. *TP53*-mutated populations were 21.7% vs. 4.0%, 7.7% vs. 0%, and 11.5% vs. 0%, *p* = 0.003, 0.042, and 0.011, respectively) Patterns in the distribution of *ASXL1*,* BCOR*, and *KRAS* mutations were similar (Table 2 and Supplementary Table S3). Since *TP53* mutation is enriched in a subset of t-AML, we further investigated the distribution of genetic mutations according to the presence of previous therapy. *TP53* mutation was enriched in t-AML group than in de novo group (23.9% vs. 12.1%, *p* = 0.032) (Supplementary Table S4). It was noted that certain mutations associated with an antecedent history of myeloproliferative neoplasm, such as the *JAK2* mutation were more commonly observed in t-AML group (8.7% in the t-AML group vs. 1.6% in *de novo* AML group). Interestingly, *DNMT3A* mutations were significantly less prevalent in the t-AML group, being found in 6.8% of t-AML cases compared to 19.5% in de novo AML group (Supplementary Table S5), which may reflect differences in the mutational landscape between therapy-related AML and de novo AML.

### *TP53* mutations in AML

**Fig. 2:**
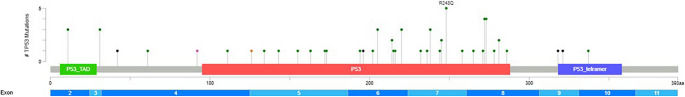
*Lollipop plot of *TP53* mutations

Additionally, when examining the characteristics of *TP53* mutations in detail, it was found that out of 50 patients, 44 (88.0%) exhibited missense substitutions, while frameshift insertions and deletion were observed in 4 patients. Transition of guanine to adenine was the most common type of *TP53* mutation (21/50, 42%), the transition from adenine to guanine was observed in 7/50 (14%). Missense mutations were categorized as disruptive mutations in 6 patients (12%), while mutations in the other 38 patients (76%) were categorized as non-disruptive mutations (see Supplementary Table S6) [21]. A lollipop plot showed that most TP53 mutations were located within the DNA-binding domain of the TP53 protein, although the overall distribution appeared relatively heterogeneous (Fig. 2). The specific details of these mutational characteristics are described in Supplementary Table S7.

### Treatment outcomes of *TP53*-mutated AML

In terms of treatment efficacy, *TP53* mutations were associated with significantly lower composite complete response (cCR) rates, consistent with previous studies. Among patients whose response could be evaluated, 183 of the 281 patients without *TP53* mutations (65.1%) achieved cCR, compared to 16 of the 48 patients with *TP53* mutations (33.3%) (*p* < 0.001) (see Table 3). When considering the intensity of treatment regimens in *TP53* wild-type AML, a higher cCR rate was observed with intensive regimens (75%) compared to less intensive regimens (45%) (*p* < 0.001). In *TP53*-mutated AML, cCR rates were also higher with intensive regimens (41.9%) versus less intensive regimens (17.6%) (*p* < 0.001) (see Supplementary Table S8).

**Table 3:** *Treatment results

	N (%)	Total (*N* = 329)	*TP53* wild-type (*N* = 281)	*TP53* mutation (*N* = 48)	*P* value
Induction result	Composite complete response	199 (60.5)	183 (65.1)	16 (33.3)	< 0.001
	Partial response	14 (4.3)	9 (3.2)	5 (10.4)	
	Persistence	74 (22.5)	55 (19.6)	19 (39.6)	
	Not evaluated	42 (12.8)	39 (12.1)	10 (19.0)	
Allogenic stem cell transplantation	No alloSCT	196 (58.3)	165 (57.7)	31 (62.0)	0.569
	AlloSCT	140 (41.7)	121 (42.3)	19 (38.0)	
Results of Allogenic stem cell transplantation	Complete response	113 (84.3)	100 (86.2)	13 (72.2)	0.303
	Engraftment failure	5 (3.7)	4 (3.4)	1 (5.6)	
	Persistence	16 (11.9)	12 (10.3)	4 (22.2)	
Relapse	Relapsed disease	110 (32.7)	93 (32.5)	17 (34.0)	0.007
	Persistent disease	88 (26.2)	67 (23.4)	21 (42.0)	

*N* number; *AlloSCT* allogenic stem cell transplantation

**Fig. 3:**
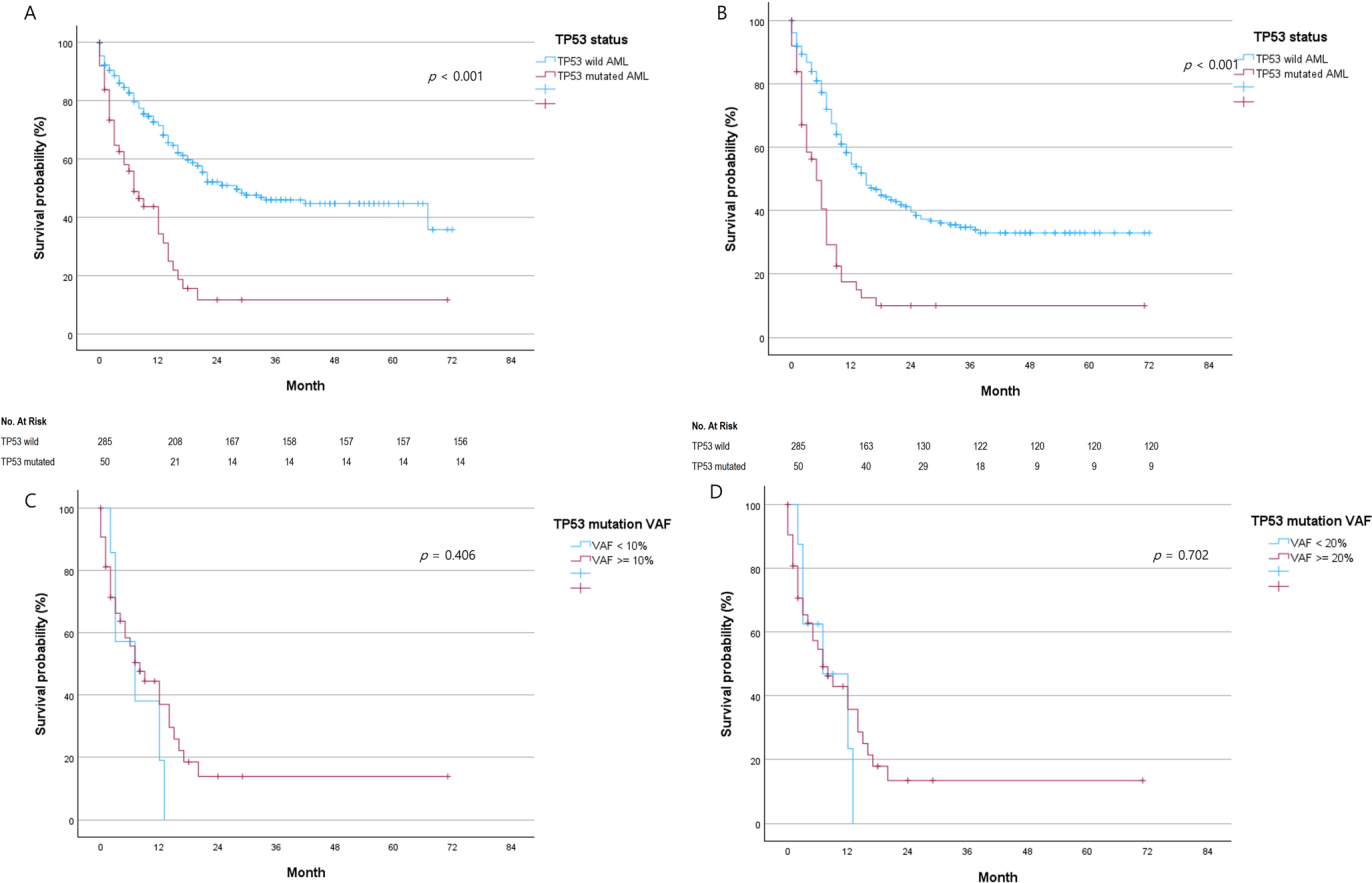
*Treatment outcomes according to *TP53* mutation. (**A**) Overall survival. (**B**) Progression-free survival. (**C**) Overall survival according to VAF (10%). (**D**) Overall survival according to VAF (20%). VAF: variant allele frequency

Kaplan–Meier survival analysis showed that patients with *TP53* mutations had significantly shorter OS than those with TP53 wild-type AML. The median OS was 7.0 months (95% CI: 3.3–10.57) in the *TP53*-mutated group compared with 28.0 months (95% CI: 15.09–40.91) in the *TP53* wild-type group (*p* < 0.001) (Fig. 3A). PFS was also significantly shorter in patients with *TP53* mutations, with a median PFS of 5.0 months (95% CI: 3.18–6.82) compared with 15.0 months (95% CI: 11.83–18.17) in the *TP53* wild-type group (*p* < 0.001) (Fig. 3B).

Differing from previous studies, an examination of OS based on *TP53* VAF values showed no significant differences when compared using cutoffs of 10% and 20% (see Fig. 3C and D). When comparing median OS based on *TP53* mutational details, it was challenging to draw statistical significance due to the small sample sizes. When comparing median OS according to TP53 mutation types, no statistically significant differences were observed among frameshift, missense, nonsense, and splicing mutations, although the small sample sizes limited the ability to detect potential differences. Additionally, the median OS for frameshift, missense, nonsense and splicing mutations did not show statistically significant differences (see Supplementary Fig. 2).

## Discussion

*TP53* mutations in AML are linked to increased genomic instability, complex cytogenetic abnormalities, advanced age, chemoresistance, and poor clinical outcomes. These mutations are commonly detected in t-AML and AML-MRC. A study found that *TP53* alterations occur in approximately 5% to 15% of de novo AML patients, but this frequency rises to 25% to 40% in therapy-related or relapsed cases. The most common type of *TP53* mutation identified is missense mutations [11, 22]. Our research also showed that *TP53* mutations were more frequently found in t-AML, with missense mutations being the most commonly identified mutation type. Previous studies have established that complex karyotype abnormalities are more prevalent in AML cases with *TP53* mutations. Our research corroborated these findings, revealing that complex karyotypes were more frequently identified in *TP53*-mutated AML compared to TP53 wild-type AML. In contrast, *TP53* wild-type AML frequently exhibited normal karyotypes or cytogenetic abnormalities that were not classified as either favorable or adverse. Furthermore, both OS and treatment response indicated poor prognosis, consistent with earlier studies. While our findings align with earlier studies, we also uncovered new insights that we believe are meaningful.

Immunophenotyping in AML cells involves analyzing surface markers to determine lineage and maturation in myeloid cells, which is crucial for identifying phenotypic changes in leukemic cells. This technique is essential for diagnosing AML, as it helps classify the disease based on various antigen categories, including precursors and myeloid markers. Additionally, immunophenotyping supports treatment response evaluation and the detection of minimal residual disease (MRD) through antigen monitoring during and after therapy [23]. While immunophenotyping is very helpful in the differential diagnosis of acute leukemia at initial diagnosis, the relationship between genetics and immunophenotype has not been clearly established. In our study, we analyzed the relationship between *TP53* mutations and the aberrant expression of various cell lineages, including B cells, T cells, and NK cells, but we could not confirm statistical significance (Table 2). Previous studies have reported that *TP53*-mutated AML is associated with lower leukocyte counts and higher CD34 expression [24]. Our findings are consistent with these observations. In contrast, we additionally observed enrichment of CD41 expression in *TP53*-mutated AML, suggesting a more primitive stem/progenitor phenotype compared to *TP53* wild-type AML.

*TP53* mutations have also been reported in non–Down syndrome acute megakaryoblastic leukemia (AMKL), where CD41 expression is commonly observed [25]. However, AMKL cases were rare in our cohort (5 of 336 patients) and were not overrepresented in the *TP53*-mutated group. Therefore, the increased frequency of CD41 expression observed in *TP53*-mutated AML in our study cannot be explained solely by the presence of AMKL. Further research is needed to explore the relationship between immunophenotyping and genetic abnormalities.

Our analysis of the NGS panel revealed that *TP53* mutations frequently occurred in isolation, with *TP53*-mutated AML patients exhibiting fewer concurrent mutations in other genes compared to those without *TP53* mutations. Genes like *CEBPA*, *FLT3*, *NPM1*,* KRAS* and *IDH1* were less frequently co-mutated with *TP53*. *TP53* mutations were known to be commonly associated with a complex aberrant karyotype, and this finding is consistent with previous reports showing that TP53-mutated AML often harbors fewer co-occurring driver mutations [26]. While the reasons for this observation remain unclear, it appears that in cases of *TP53* mutations, cytogenetic alterations are more prevalent than changes at the genetic level within individual genes. This finding suggests a need for further research to explore the underlying mechanisms and implications of these cytogenetic changes in relation to *TP53* mutations. Furthermore, certain mutations, such as *DNMT3A*, were less frequent in the t-AML group, while *TP53* mutations were observed in 14.9% of t-AML patients. Based on the NGS findings, further research into t-AML could provide valuable insights for understanding prognosis and treatment planning for t-AML patients, emphasizing the need for additional studies in this area. Previous studies have demonstrated that *TP53*-mutated AML is strongly associated with complex karyotypes and tends to harbor fewer co-occurring driver mutations [14, 27]. Our findings are consistent with these reports.

In our cohort, we further examined the presence of multiple *TP53* mutations as a proxy for multi-hit alterations. Among 50 patients with *TP53*-mutated AML, 9 harbored two or more *TP53* mutations. Complex karyotype was observed in 7 of 8 evaluable patients with ≥ 2 *TP53* mutations (87.5%) and in 28 of 41 patients with a single *TP53* mutation (68.3%), suggesting that complex cytogenetic abnormalities were frequent in both groups and numerically more common in patients with multiple *TP53* mutations. However, because information on copy-neutral loss of heterozygosity or other second-hit alterations was not systematically available, the allelic configuration of *TP53* mutations could not be definitively determined in this cohort.

From a treatment perspective, *TP53* mutations were associated with significantly poorer prognosis, consistent with prior studies. In both *TP53* wild-type and *TP53*-mutated AML, higher cCR rates were observed with intensive treatment regimens compared to less intensive regimens. We also examined treatment outcomes according to treatment intensity. Patients who received less-intensive regimens showed lower complete response rates compared with those who received intensive chemotherapy, regardless of TP53 mutation status. These findings should be interpreted with caution, as treatment intensity may be influenced by clinical factors such as age, comorbidities, or performance status. In this study, an analysis of survival data revealed that OS and PFS were poor for patients with *TP53* mutations, consistent with previous research. However, there was no significant difference in outcomes based on VAF values. Even when analyzing thresholds of 10% and 20%, no differences were observed, suggesting that further research is needed to determine whether VAF values have an impact on clinical outcomes. Previous studies have suggested that higher *TP53* VAF may be associated with poorer prognosis in *TP53*-mutated AML. In our cohort, however, we did not observe a significant association between *TP53* VAF and survival outcomes when applying cutoffs of 10% and 20%. Recent studies have suggested that the prognostic impact of *TP53* alterations may depend more strongly on allelic configuration, such as multi-hit versus single-hit *TP53*, rather than VAF alone [22, 28]. In this context, our findings may support the concept that VAF alone may not fully capture the biological complexity of *TP53*-mutated AML.

*TP53*-mutated AML harbors a more primitive stem/progenitor cell-like nature and exhibits a higher degree of leukocytopenia in peripheral blood. *TP53*-mutated AML is unequivocally linked to poor prognosis and worse treatment outcomes, underscoring the importance of understanding the unique characteristics of this mutation in the context of AML. In this study, we aimed to provide a more detailed characterization of *TP53*-mutated AML, exploring its association with genomic instability, complex cytogenetic abnormalities, and treatment response patterns. Moreover, while we established that *TP53* mutations often occur in isolation, further investigation into the interplay between genetic alterations and cytogenetic changes will be crucial in understanding the underlying mechanisms driving poor outcomes. This study highlights the necessity for additional research that can refine our knowledge of *TP53*-mutated AML, ultimately guiding clinical practice toward tailored therapeutic strategies.

## Supplementary Information

Below is the link to the electronic supplementary material.

Supplementary Material 1

Supplementary Material 2: Peripheral blood blasts according to TP53 mutation

Supplementary Material 3: Median overall survival according to TP53 mutational details

## Data Availability

The data that support the findings of this study are available from the corresponding author upon reasonable request. All relevant raw data, as described in the manuscript, will be made freely available to any researcher wishing to use them for non-commercial purposes, without breaching participant confidentiality.

## Declarations

### Use of artificial intelligence tools

During the preparation of this work, the authors used artificial intelligence-assisted tools to enhance the readability and language of the manuscript. After utilizing these tools, the authors thoroughly reviewed and edited the content as necessary, and they take full responsibility for the final publication.
