# Supplementary material for: Distinct immunophenotypic and clinical features of TP53-mutated acute myeloid leukemia: high CD34/CD41 expression and lower leukocyte counts
Source: Ann Hematol. 2026 Apr 21;105(5):259. doi: 10.1007/s00277-026-07001-4 (PMC13095964; doi:10.1007/s00277-026-07001-4)
Supplement: Supplementary file 1 — Supplementary Material 1 [file 277_2026_7001_MOESM1_ESM.docx]

Supplementary Table 1. Next-generation sequencing (NGS) panel list.

| SNUH | 103 genes (from 2021) | All coding exons | *APC, ARID1B, ARID2, ASXL1, ATM, B2M, BCOR, BCORL1, CARD11, CCND1, CD28, CD58, CD79A, CHD8, CREBBP, CUX1, DDX41, DIS3, DNMT3A, EP300, ETV6, EZH2, FBXW7, GATA2, GNA13, IKZF1, KDM6A, KIT, KMT2A, KMT2D, LUC7L2, MYC, NF1, NRAS, PHF6, PTEN, RAD21, RB1, RUNX1, SH2B3, SMC1A, SMC3, SRSF2, STAG2, TET2, TNFAIP3, TNFRSF14, TP53, TRAF3, U2AF2, WT1, ZAP70, ZRSR2* |
| --- | --- | --- | --- |
|  |  | Hot spots | *ABL1, ANKRD26, ATRX, BIRC3, BRAF, BTK, CALR, CBL, CBLB, CBLC, CCND2, CDC25C, CDKN2A, CEBPA, CSF3R, CXCR4, DCK, DHX15, ETNK1, FLT3, FOXO1, GATA1, GNAS, HRAS, IDH1, IDH2, JAK2, JAK3, KMT2C, KRAS, MAP2K1, MEF2B, MPL, MYD88, NOTCH1, NOTCH2, NPM1, PDGFRA, PPM1D, PTPN11, RBBP6, RHOA, RPS14, SETBP1, SF3B1, SLC29A1, STAT3, STAT5B, U2AF1, XPO1* |
|  | 76 genes (2017-2020) | | *ABL1, AKT1, ALK, ASXL1, ATM, BCL2, BCR, BRAF, BTK, CALR, CBFB, CDK4, CDK6, CDKN2A, CEBPA, CREBBP, CSF1, CSF3R, DDX3X, DNMT3A, EGFR, EP300, ERBB2, ETV6, EZH2, FGFR1, FGFR2, FGFR3, FGFR4, FLT1, FLT3, FLT4, IDH1, IDH2, IGF1R, IKZF1, JAK2, KDR, KIT, KMT2A, KRAS, MAP2K1, MET, MPL, MTOR, MYD88, NFKB2, NPM1, NRAS, NTRK1, PBX1, PDGFRA, PDGFRB, PIK3CA, PIK3R1, PML, PRKCB, PTCH1, PTEN, PTPN11, RARA, RB1, RET, ROS1, RPS15, RUNX1, SETBP1, SF3B1, SMO, SRSF2, SYK, TET2, TNFAIP3, TP53, TSC1, TSC2* |
| SNUBH | 38 genes | | *ASXL1, BCOR, BRAF, CALR, CBL, CEBPA, CSF3R, DNMT3A, ETV6, EZH2, FLT3, GATA2, HRAS, IDH1, IDH2, IKZF1, JAK2, KIT, KRAS, MPL, MYD88, NF1, NPM1, NRAS, PHF6, PRPF8, PTPN11, RUXN1, SETBP1, SF3B1, SH2B3, SRSF2, STAG2, TET2, TP53, U2AF1, WT1, and ZRSR2* |

Supplementary Table 2. Immunophenotyping

|  |  | N (%) | Total  (N=336) | No *TP53*  (N=286) | *TP53* mutation  (N=50) | *p* value |
| --- | --- | --- | --- | --- | --- | --- |
| Cytoplasmic marker | TdT | negative | 306 (92.2) | 258 (91.2) | 48 (98.0) | 0.102 |
|  |  | positive | 26 (7.8) | 25 (8.8) | 1 (2.0) |  |
|  | MPO | negative | 75 (22.6) | 62 (21.9) | 13 (26.5) | 0.475 |
|  |  | positive | 257 (77.4) | 221 (78.1) | 36 (73.5) |  |
|  | cytCD79a | negative | 265 (81.3) | 223 (80.2) | 42 (87.5) | 0.232 |
|  |  | positive | 61 (18.7) | 55 (19.8) | 6 (12.5) |  |
|  | cytCD3 | negative | 324 (97.6) | 277 (97.9) | 47 (95.9) | 0.408 |
|  |  | Positive | 8 (2.4) | 6 (2.1) | 2 (4.1) |  |
|  | cytIgM | negative | 152 (81.7) | 129 (82.7) | 23 (76.7) | 0.434 |
|  |  | positive | 34 (18.3) | 27 (17.3) | 7 (23.3) |  |
|  | cytCD22 | negative | 185 (98.4) | 157 (100) | 28 (90.3) | <0.001 |
|  |  | positive | 3 (1.6) | 0 | 3 (9.7) |  |
|  | CD2 | negative | 239 (97.2) | 202 (97.1) | 37 (97.4) | 0.931 |
|  |  | positive | 7 (2.8) | 6 (2.9) | 1 (2.6) |  |
|  | CD3 | negative | 228 (98.7) | 192 (99.5) | 36 (94.7) | 0.018 |
|  |  | positive | 3 (1.3) | 1 (0.5) | 2 (5.3) |  |
|  | CD5 | negative | 169 (88.5) | 143 (90.5) | 26 (78.8) | 0.055 |
|  |  | positive | 22 (11.5) | 15 (9.5) | 7 (21.2) |  |
|  | CD7 | negative | 229 (69.0) | 195 (69.1) | 34 (68.0) | 0.871 |
|  |  | positive | 103 (31.0) | 87 (30.9) | 16 (32.0) |  |
|  | CD10 | negative | 237 (95.2) | 198 (94.7) | 39 (97.5) | 0.455 |
|  |  | positive | 12 (4.8) | 11 (5.3) | 1 (2.5) |  |
|  | CD11c | negative | 17 (13.2) | 14 (12.5) | 3 (17.6) | 0.559 |
|  |  | positive | 112 (86.8) | 98 (87.5) | 14 (82.4) |  |
|  | CD13 | negative | 44 (13.3) | 36 (12.7) | 8 (16.3) | 0.492 |
|  |  | positive | 288 (86.7) | 247 (87.3) | 41 (83.7) |  |
|  | CD14 | negative | 216 (83.7) | 191 (84.5) | 25 (78.1) | 0.360 |
|  |  | positive | 42 (16.3) | 35 (15.5) | 7 (21.9) |  |
|  | CD19 | negative | 308 (92.5) | 260 (91.9) | 48 (96.0) | 0.307 |
|  |  | positive | 25 (7.5) | 23 (8.1) | 2 (4.0) |  |
|  | CD20 | negative | 256 (98.8) | 215 (98.6) | 41 (100) | 0.450 |
|  |  | positive | 3 (1.2) | 3 (1.4) | 0 |  |
|  | CD33 | negative | 10 (3.0) | 8 (2.8) | 2 (4.1) | 0.635 |
|  |  | positive | 322 (97.0) | 275 (97.2) | 47 (95.9) |  |
|  | CD34 | negative | 97 (29.2) | 89 (31.4) | 8 (16.3) | 0.032 |
|  |  | positive | 235 (70.8) | 194 (68.6) | 41 (83.7) |  |
|  | CD41 | negative | 180 (95.2) | 153 (97.5) | 27 (84.4) | 0.002 |
|  |  | positive | 9 (4.8) | 4 (2.5) | 5 (15.6) |  |
|  | CD56 | negative | 218 (79.9) | 181 (78.7) | 37 (86.0) | 0.270 |
|  |  | positive | 55 (20.1) | 49 (21.3) | 6 (14.0) |  |
|  | CD64 | negative | 105 (45.5) | 90 (44.6) | 15 (51.7) | 0.468 |
|  |  | positive | 126 (54.5) | 112 (55.4) | 14 (48.3) |  |
|  | CD117 | negative | 43 (13.0) | 38 (13.4) | 5 (10.2) | 0.535 |
|  |  | positive | 289 (87.0) | 245 (86.6) | 44 (89.8) |  |
|  | HLA-DR | negative | 19 (12.8) | 15 (11.6) | 4 (21.1) | 0.252 |
|  |  | positive | 129 (87.2) | 114 (88.4) | 15 (78.9) |  |

Supplementary Table 3. Next-generation sequencing list

|  | Total | No *TP53* | *TP53* mutation | *P* value |
| --- | --- | --- | --- | --- |
| *ASXL1* | 54 (16.1) | 49 (17.1) | 5 (10.0) | 0.205 |
| *BCOR* | 14 (4.2) | 14 (4.9) | 0 | 0.110 |
| *BRAF* | 3 (0.9) | 3 (1.0) | 0 | 0.467 |
| *CARL* | 3 (0.9) | 3 (1.0) | 0 | 0.467 |
| *CBL* | 4 (1.2) | 4 (1.4) | 0 | 0.400 |
| *CEBPA* | 33 (9.8) | 33 (11.5) | 0 | 0.011 |
| *CSF3R* | 10 (3.0) | 10 (3.5) | 0 | 0.179 |
| *DNMT3A* | 63 (18.8) | 55 (19.2) | 8 (16.0) | 0.589 |
| *ETV6* | 11 (3.3) | 9 (3.1) | 2 (4.0) | 0.754 |
| *EZH2* | 11 (3.3) | 8 (2.8) | 3 (6.0) | 0.240 |
| *FLT3* | 18 (5.4) | 18 (6.3) | 0 | 0.068 |
| *GATA2* | 11 (3.3) | 9 (3.1) | 2 (4.0) | 0.754 |
| *IDH1* | 22 (6.5) | 22 (7.7) | 0 | 0.042 |
| *IDH2* | 36 (10.7) | 33 (11.5) | 3 (6.0) | 0.243 |
| *IKZF1* | 11 (3.3) | 10 (3.5) | 1 (2.0) | 0.583 |
| *JAK2* | 9 (2.7) | 7 (2.4) | 2 (4.0) | 0.530 |
| *KIT* | 21 (6.3) | 20 (7.0) | 1 (2.0) | 0.178 |
| *KRAS* | 18 (5.4) | 18 (6.3) | 0 | 0.068 |
| *MPL* | 5 (1.5) | 4 (1.4) | 1 (2.0) | 0.746 |
| *MYD88* | 1 (0.3) | 1 (0.3) | 0 | 0.675 |
| *NF1* | 6 (1.8) | 4 (1.4) | 2 (4.0) | 0.200 |
| *NPM1* | 64 (19.0) | 62 (21.7) | 2 (4.0) | 0.003 |
| *NRAS* | 47 (14.0) | 38 (13.3) | 9 (18.0) | 0.375 |
| *PHF6* | 6 (1.8) | 5 (1.7) | 1 (2.0) | 0.901 |
| *PTPN11* | 24 (7.1) | 18 (6.3) | 6 (12.0) | 0.148 |
| *RUNX1* | 46 (13.7) | 40 (14.0) | 6 (12.0) | 0.706 |
| *SETBP1* | 9 (2.7) | 8 (2.8) | 1 (2.0) | 0.747 |
| *SF3B1* | 10 (3.0) | 9 (3.1) | 1 (2.0) | 0.660 |
| *SH2B3* | 9 (2.7) | 8 (2.8) | 1 (2.0) | 0.747 |
| *SRSF2* | 27 (8.0) | 25 (8.7) | 2 (4.0) | 0.255 |
| *STAG2* | 13 (3.9) | 12 (4.2) | 1 (2.0) | 0.458 |
| *TET2* | 62 (18.5) | 53 (18.5) | 9 (18.0) | 0.929 |
| *U2AF1* | 15 (4.5) | 13 (4.5) | 2 (4.0) | 0.863 |
| *WT1* | 14 (4.2) | 12 (4.2) | 2 (4.0) | 0.949 |
| *ZRSR2* | 4 (1.2) | 4 (1.4) | 0 | 0.400 |

Supplementary Table 4. Secondary etiology

|  | De novo  (N=257) | Treatment related  (N=46) | *p* value |
| --- | --- | --- | --- |
| No *TP53*  (N=286) | 226 (87.9) | 35 (76.1) | 0.032 |
| *TP53* mutation  (N=50) | 31 (12.1) | 11 (23.9) |  |

Supplementary Table 5. Genetic characteristics of therapy-related acute myeloid leukemia

|  | De novo AML  (N=257) | Therapy-related AML  (N=46) | *P* value |
| --- | --- | --- | --- |
| *ASXL1* | 37 ( 14.4) | 8 (17.4) | 0.599 |
| *BCR* | 16 (6.2) | 4 (8.7) | 0.534 |
| *CEBPA* | 30 (11.7) | 2 (4.3) | 0.137 |
| *DNMT3A* | 50 (19.5) | 3 (6.5) | 0.033 |
| *FLT3* | 15 (5.8) | 2 (4.3) | 0.686 |
| *IDH1* | 18 (7.0) | 4 (8.7) | 0.684 |
| *IDH2* | 30 (11.7) | 3 (6.5) | 0.302 |
| *IKZF1* | 6 (2.3) | 2 (4.3) | 0.433 |
| *JAK2* | 4 (1.6) | 4 (8.7) | 0.005 |
| *KIT* | 20 (7.8) | 1 (2.2) | 0.168 |
| *KRAS* | 14 (5.4) | 3 (6.5) | 0.771 |
| *MPL* | 2 (0.8) | 2 (4.3) | 0.051 |
| *NPM1* | 56 (21.8) | 4 (8.7) | 0.040 |
| *NRAS* | 37 (14.4) | 3 (6.57) | 0.146 |
| *PTPN11* | 19 (7.4) | 5 (10.9) | 0.421 |
| *RUNX1* | 31 (12.1) | 8 (17.4) | 0.166 |
| *SETBP1* | 3 (1.2) | 3 (6.5) | 0.016 |
| *SF3B1* | 4 (1.6) | 3 (6.5) | 0.039 |
| *SRSF2* | 20 (7.8) | 2 (4.3) | 0.408 |
| *STAG2* | 12 (4.7) | 0 | 0.135 |
| *TET2* | 43 (16.7) | 9 (19.6) | 0.639 |
| *TP53* | 31 (12.1) | 11 (23.9) | 0.032 |
| *U2AF1* | 7 (2.7) | 2 (4.3) | 0.550 |
| *WT1* | 10 (3.9) | 0 | 0.174 |
| *ZRSR2* | 3 (1.2) | 1 (2.2) | 0.582 |

Supplementary Table 6. Mutational details of *TP53* mutation

| Types of mutation | N (%) | *TP53* mutation  (N=50) | 2^nd^ *TP53* mutation  (N=9) | 3^rd^ *TP53* mutation  (N=2) |
| --- | --- | --- | --- | --- |
| Missense substitutions | Total | 44 (88) | 8 (88.9) | 2 (100) |
|  | Transition  C>T  T>C | 3  0 | 0  1 |  |
|  | Transition  A>G  G>A | 7  21 | 2  4 |  |
|  | Transversion  C>G  C>A | 2  0 |  |  |
|  | Transversion  T>G  T>A | 4  0 | 1  0 | 1  1 |
|  | Transversion  G>C  G>T | 4  2 |  |  |
|  | Transversion  A>C  A>T | 1  0 |  |  |
|  | Disruptive mutation | 6 | 1 | 0 |
|  | Non-disruptive mutation | 38 | 7 | 2 |
| Frameshift insertion/deletion | | 4 (8) | 1 (11.1) | 0 |
| Non-sense mutation | | 1 (2) | 0 | 0 |
| Splicing mutation | | 1 (2) | 0 | 0 |

Supplementary Table 7. Mutational details of *TP53* mutation

|  | AA | CDS | VAF(%) | Disruptive | Oncogenic |
| --- | --- | --- | --- | --- | --- |
| 1 | p.Leu265Arg | c.794T>G | 63.9 | Non-disruptive | Likely Oncogenic |
| 2 | p.Val272Met | c.427G>A | 9.61 | Non-disruptive | Likely Oncogenic |
| 3 | p.Arg273Cys | c.817C>T | 85.6 | Non-disruptive | Likely Oncogenic |
| 4 | p.Glu271Lys | c.811G>A | 5.2 | Non-disruptive | Likely Oncogenic |
|  | p.Tyr236Cys | c.707A>G | 72.31 | Non-disruptive | Likely Oncogenic |
|  | p.Phe134Cys | c.401T>G | 7.17 | Non-disruptive | Likely Oncogenic |
| 5 | p.Asp281Glu | c.843C>G | 21.78 | Non-disruptive | Inconclusive |
|  | p.Tyr220His | c.658T>C | 21.47 | Non-disruptive | Likely Oncogenic |
| 6 | p.His214Profs*33 | c.641_642delinsC | 21.8 | Disruptive | Likely Oncogenic |
| 7 | p.Glu258Gly | c.773A>G | 81.7 | Non-disruptive | Likely Oncogenic |
| 8 | p.Glu11Gln | c.31G>C | 52.9 | Non-disruptive | Unknown |
| 9 | p.Arg196* | c.586C>T | 23.24 | Disruptive | Likely Oncogenic |
| 10 | p.Leu93Phefs*50 | c.277_293del | 70.8 | Disruptive | Likely Oncogenic |
| 11 | p.Pro318Glnfs*27 | c.953del | 62.24 | Disruptive | Likely Oncogenic |
| 12 | p.Gly245Asp | c.734G>A | 2.85 | Disruptive | Likely Oncogenic |
| 13 | p.Val272Met | c.814G>A | 88.35 | Non-disruptive | Likely Oncogenic |
| 14 | p.Arg248Gln | c.743G>A | 91.41 | Disruptive | Likely Oncogenic |
| 15 | p.Ile232Ser | c.695T>G | 84.1 | Non-disruptive | Likely Oncogenic |
| 16 | p.Arg248Gln | c.743G>A | 85.9 | Disruptive | Likely Oncogenic |
| 17 | p.Lys321Asnfs*24 | c.960del | 37.1 | Disruptive | Likely Oncogenic |
|  | p.Cys238Tyr | c.713G>A | 24.3 | Non-disruptive | Likely Oncogenic |
| 18 | p.Asp281Gly | c.842A>G | 2.7 | Non-disruptive | Inconclusive |
| 19 | p.Tyr220Cys | c.659A>G | 51.4 | Non-disruptive | Oncogenic |
| 20 | p.Thr155Pro | c.463A>C | 3.1 | Non-disruptive | Likely Oncogenic |
| 21 | p.His214Arg | c.641A>G | 70.1 | Non-disruptive | Likely Oncogenic |
| 22 | p.Tyr205Asp | c.613T>G | 52.8 | Non-disruptive | Likely Oncogenic |
| 23 | p.Arg202His | c.605G>A | 50.6 | Non-disruptive | Likely Oncogenic |
| 24 | p.Tyr220Cys | c.659A>G | 85.1 | Non-disruptive | Oncogenic |
| 25 | p.Arg337Cys | c.1009C>T | 40.9 | Non-disruptive | Likely Oncogenic |
| 26 | p.Gly245Ser | c.733G>A | 52.7 | Non-disruptive | Oncogenic |
|  | p.Cys238Tyr | c.713G>A | 44.6 | Non-disruptive | Likely Oncogenic |
| 27 | splicing | c.376-2A>G | 42 | Disruptive | Likely Oncogenic |
| 28 | p.Arg273Cys | c.817C>T | 8.7 | Non-disruptive | Likely Oncogenic |
| 29 | p.Val172Phe | c.514G>T | 5.4 | Non-disruptive | Likely Oncogenic |
|  | p.Val143Met | c.427G>A | 7.6 | Non-disruptive | Likely Oncogenic |
|  | p.Leu111Gln | c.332T>A | 10.2 | Non-disruptive | Likely Oncogenic |
| 30 | p.Asp61Tyr | c.181G>T | 18.5 | Non-disruptive | Unknown |
| 31 | p.Tyr205Asp | c.613T>G | 22.9 | Non-disruptive | Likely Oncogenic |
| 32 | p.Arg273His | c.818G>A | 51.1 | Non-disruptive | Oncogenic |
| 33 | p.Val272Met | c.814G>A | 47.8 | Non-disruptive | Likely Oncogenic |
|  | p.Tyr205Cys | c.614A>G | 48.9 | Non-disruptive | Likely Oncogenic |
| 34 | p.Cys238Tyr | c.713G>A | 43.77 | Non-disruptive | Likely Oncogenic |
|  | p.Ser215Asn | c.644G>A | 43.27 | Non-disruptive | Likely Oncogenic |
| 35 | p.Pro278Arg | c.833C>G | 4.4 | Non-disruptive | Likely Oncogenic |
| 36 | p.Arg248Gln | c.743G>A | 62.3 | Disruptive | Likely Oncogenic |
| 37 | p.Val31Ile | c.91G>A | 50.58 | Non-disruptive | Unknown |
| 38 | p.Tyr163Cys | c.488A>G | 41.75 | Non-disruptive | Likely Oncogenic |
| 39 | p.Glu11Gln | c.31G>C | 45.9 | Non-disruptive | Unknown |
| 40 | p.Arg248Gln | c.743G>A | 57.45 | Disruptive | Likely Oncogenic |
| 41 | p.Val31Ile | c.91G>A | 48.32 | Non-disruptive | Unknown |
| 42 | p.Glu11Gln | c.31G>C | 50.9 | Non-disruptive | Unknown |
| 43 | p.Glu286Gly | c.857A>G | 12 | Non-disruptive | Likely Oncogenic |
|  | p.Asp42Valfs*2 | c.125del | 9.81 | Disruptive | Likely Oncogenic |
| 44 | p.Gly244Ser | c.730G>A | 56.3 | Non-disruptive | Oncogenic |
| 45 | p.Arg248Gln | c.743G>A | 95 | Disruptive | Likely Oncogenic |
| 46 | p.Val272Met | c.814G>A | 39.15 | Non-disruptive | Likely Oncogenic |
|  | p.Leu194Arg | c.581T>G | 41.65 | Disruptive | Likely Oncogenic |
| 47 | p.Val31Ile | c.91G>A | 47.55 | Non-disruptive | Unknown |
| 48 | p.Val173Leu | c.517G>C | 64.7 | Non-disruptive | Likely Oncogenic |
| 49 | p.Val216Met | c.646G>A | 50.05 | Non-disruptive | Likely Oncogenic |
| 50 | p.Arg273His | c.818G>A | 59.35 | Non-disruptive | Oncogenic |

Supplementary Table 8. Treatment result according to intensiveness of induction

|  | | Intensive regimen  (N=219) | | *p* value | Less intensive regimen  (N=110) | | *p* value |
| --- | --- | --- | --- | --- | --- | --- | --- |
|  |  | *TP53*-WT  (N=188) | *TP53*-mutated  (N=31) |  | *TP53*-WT  (N=93) | *TP53*-mutated  (N=17) |  |
| Induction result | Composite CR | 141 (75.0) | 13 (41.9) | < 0.001 | 42 (45.2) | 3 (17.6) | < 0.001 |
|  | PR | 5 (2,7) | 1 (3.2) |  | 4 (4.3) | 4 (23.5) |  |
|  | Persistence | 35 (18.6) | 16 (51.6) |  | 20 (21.5) | 3 (17.6) |  |
|  | N/E | 7 (3.7) | 1 (3.2) |  | 27 (29.0) | 7 (41.2) |  |

Supplementary Figure 1. Peripheral blood blasts according to TP53 mutation

Supplementary figure 2. Median overall survival according to TP53 mutational details
