# Supplementary figures and images for: Distinct immunophenotypic and clinical features of TP53-mutated acute myeloid leukemia: high CD34/CD41 expression and lower leukocyte counts
Source: Ann Hematol. 2026 Apr 21;105(5):259. doi: 10.1007/s00277-026-07001-4 (PMC13095964; doi:10.1007/s00277-026-07001-4)

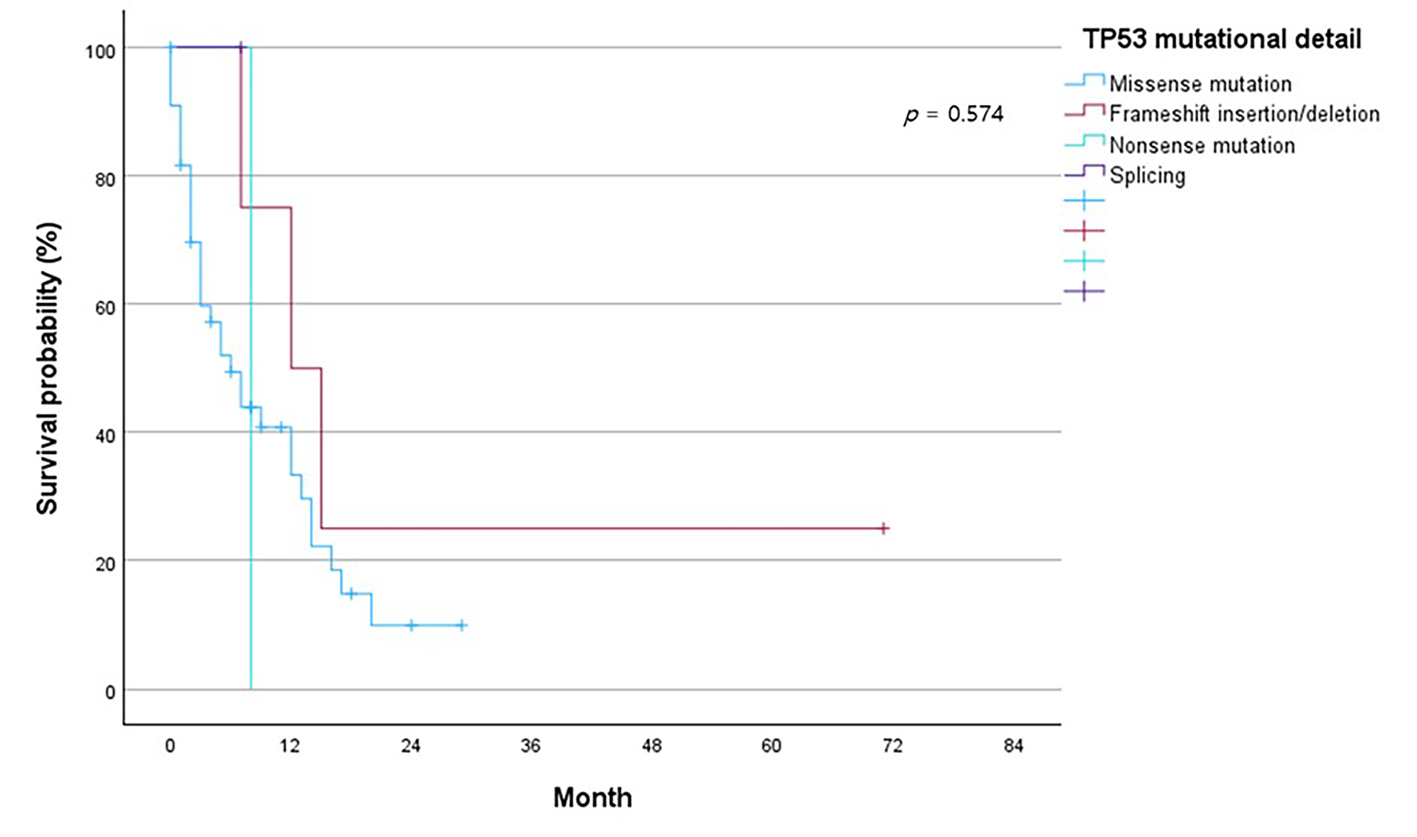

Supplement: Supplementary file 2 — Supplementary Material 2: Peripheral blood blasts according to TP53 mutation [file 277_2026_7001_MOESM2_ESM.jpg]

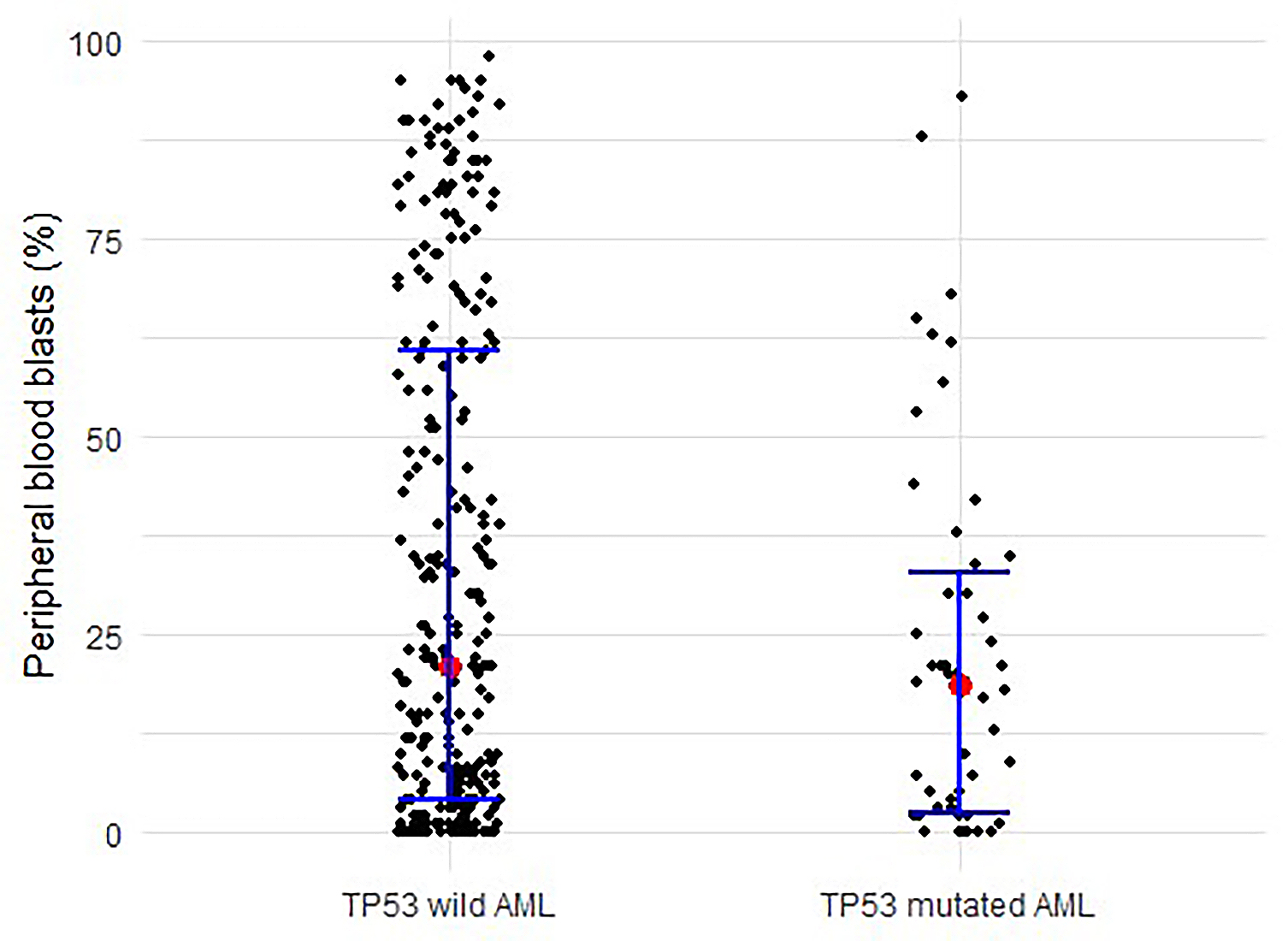

Supplement: Supplementary file 3 — Supplementary Material 3: Median overall survival according to TP53 mutational details [file 277_2026_7001_MOESM3_ESM.jpg]
